# Effects of perioperative low-dose naloxone on the immune system in patients undergoing laparoscopic-assisted total gastrectomy: a randomized controlled trial

**DOI:** 10.1186/s12871-024-02524-7

**Published:** 2024-05-08

**Authors:** Xiangzhen Min, Yan Ma, Yufang Leng, Xiaoxi Li, Jianmin Zhang, Shoucai Xu, Xiuqin Wang, Renjun Lv, Jie Guo, Huaixin Xing

**Affiliations:** 1 The First School of Clinical Medicine, Lanzhou University, Lanzhou, Gansu, China; 2 Department of Anesthesiology, Shandong Cancer Hospital and Institute Affiliated to Shandong First Medical University, Shandong Academy of Medical Science, Jinan, Shandong, China; 3 Department of Anesthesiology, Affiliated Hospital of Shandong University of Traditional Chinese Medicine, Jinanm, Shandong Province, China; 4 Department of Anesthesiology, The First Hospital of Lanzhou University, Lanzhou, Gansu, China

**Keywords:** Low-dose naloxone, Immune function, Laparoscopic-assisted total gastrectomy, Postoperative complications

## Abstract

**Background:**

Low immune function after laparoscopic total gastrectomy puts patients at risk of infection-related complications. Low-dose naloxone (LDN) can improve the prognosis of patients suffering from chronic inflammatory diseases or autoimmune diseases. The use of LDN during perioperative procedures may reduce perioperative complications. The purpose of this study was to examine the effects of LDN on endogenous immune function in gastric cancer patients and its specific mechanisms through a randomized controlled trial.

**Methods:**

Fifty-five patients who underwent laparoscopic-assisted total gastrectomy were randomly assigned to either a naloxone group (*n* = 23) or a nonnaloxone group (*n* = 22). Patients in the naloxone group received 0.05 µg/kg-1.h^− 1^naloxone from 3 days before surgery to 5 days after surgery via a patient-controlled intravenous injection (PCIA) pump, and patients in the nonnaloxone group did not receive special treatment. The primary outcomes were the rates of postoperative complications and immune function assessed by NK cell, CD3^+^ T cell, CD4^+^ T cell, CD8^+^ T cell, WBC count, neutrophil percentage, and IL-6 and calcitonin levels. The secondary outcomes were the expression levels of TLR4 (Toll-like receptor), IL-6 and TNF-α in gastric cancer tissue.

**Results:**

Compared with the nonnaloxone group, the naloxone group exhibited a lower incidence of infection (in the incision, abdomen, and lungs) (*P* < 0.05). The numbers of NK cells and CD8^+^ T cells in the naloxone group were significantly greater than those in the nonnaloxone group at 24 h after surgery (*P* < 0.05) and at 96 h after surgery (*P* < 0.05). Compared with those in the nonnaloxone group, the CD3 ^+^ T-cell (*P* < 0.05) and CD4 ^+ ^T-cell (*P* < 0.01) counts were significantly lower in the naloxone group 24 h after surgery. At 24 h and 96 h after surgery, the WBC count (*P* < 0.05) and neutrophil percentage (*P* < 0.05) were significantly greater in the nonnaloxone group. The levels of IL-6 (*P* < 0.05) and calcitonin in the nonnaloxone group were significantly greater at 24 h after surgery. At 24 h following surgery, the nonnaloxone group had significantly greater levels of IL-6 (*P* < 0.05) and calcitonin than did the naloxone group. Compared with those in the naloxone group, the expression levels of TLR4 (*P* < 0.05) in gastric cancer tissue in the naloxone group were greater; however, the expression levels of IL-6 (*P* < 0.01) and TNF-α (*P* < 0.01) in the naloxone group were greater than those in the nonnaloxone group.

**Conclusion:**

Laparoscopic total gastrectomy patients can benefit from 0.05 ug/kg^− 1^. h^− 1^ naloxone by reducing their risk of infection. It is possible that LDN alters the number of cells in lymphocyte subpopulations, such as NK cells, CD3 ^+ ^T cells, and CD4^ + ^T cells, and the CD4^+^/CD8 ^+^ T-cell ratio or alters TLR4 receptor expression in immune cells, thereby altering immune cell activity.

**Trial registration:**

The trial was registered at the Chinese Clinical Trial Registry on 24/11/2023 (ChiCTR2300077948).

**Supplementary Information:**

The online version contains supplementary material available at 10.1186/s12871-024-02524-7.

## Background

Globally, the incidence and mortality of gastric cancer remain high among cancer patients. Currently, comprehensive surgical treatment has remained the most effective treatment method for gastric cancer [1]. Nevertheless, surgery is likely to result in complications such as the circulation of cancer cells into the bloodstream, dysfunction of the autonomic nervous system, and ischaemia‒reperfusion injuries [2]. Patients experience a concentration of complications during the perioperative period, which generally occurs between 5 and 7 days before surgery and 7–12 days after surgery. The incidence of complications caused by immune deficiency in gastric cancer patients was reported to be as high as 13.5% in one study [3]. One of the urgent issues that clinical doctors must address is finding the best drugs or methods for reducing perioperative immune-related complications.

Naloxone is a nonselective opiate receptor antagonist used to manage acute opioid overdose or intoxication. In recent years, unconventional uses of naloxone have been identified. Studies [4, 5] have shown that low-dose naloxone (LDN) can improve the prognosis in patients with chronic inflammatory diseases or autoimmune diseases. In addition, studies [6, 7] have suggested that the use of low-dose naloxone during surgery may reduce the risk of perioperative complications and cancer recurrence following surgery.

LDN can improve the prognosis in patients with breast cancer and gastrointestinal tumours, possibly by delaying tumour progression, interfering with cell signalling and improving immune function [8, 9]. As a result of LDN, macrophage function can improve, and proinflammatory factors can increase [10, 11]. Several studies have demonstrated that perioperative LDN administration can significantly increase CD4^+^/CD8^+^ T-cell ratios as well as the number of natural killer cells. Reports [12, 13] have suggested that naltrexone may modulate the secretion of inflammatory cytokines in response to intracellular TLR activity, suggesting that it may affect immune cell exhaustion by regulating TLRs. However, little is known about how LDN can improve the immune function of patients with gastric cancer. Therefore, the purpose of this study was to investigate the effects of LDN on endogenous immune function in gastric cancer patients and the specific underlying mechanisms by conducting a randomized controlled trial.

## Methods

This study was a randomized controlled trial that was approved by the Ethics Committee of the Cancer Hospital Affiliated with Shandong First Medical University before its initiation (SDTHEC2023003180). The study had already been registered at the China Clinical Trial Registration Center (www.chictr.org.cn, number: ChiCTR2300077948) before patient enrolment. All patients were informed of the research protocol and signed an informed consent form before enrolment.

To determine the sample size for this study, a pilot study involving 20 patients was carried out as a preliminary investigation. This study was conducted from November 24, 2023, to December 24, 2023, at the Cancer Hospital Affiliated with Shandong First Medical University. We enrolled 67 patients aged 35 to 75 years who underwent laparoscopic total gastrectomy and had American Society of Anaesthesiology (ASA) physical status II to III. Patients with severe heart or lung disease, severe liver or kidney dysfunction, an allergy to naloxone, a history of neoadjuvant chemoradiotherapy, or an opioid addiction or abuse were excluded from participation in the study. To generate a random arrangement given the number of seeds, anaesthesiologists used the SAS statistical software Proc Plan process statements. Sealed, opaque envelopes each contained a random number. This method allowed us to divide the enrolled patients into two groups: a naloxone group (*n* = 23) and a nonnaloxone group (*n* = 22). The naloxone group patients were treated with open venous channels 3 days before surgery, and 0.05 µg/kg^-1^.h^-1^ naloxone was continuously infused with a patient-controlled intravenous injection (PCIA) pump; nonnaloxone did not undergo special treatment before surgery. Two groups of patients used the same anaesthesia methods and drugs during surgery, and to reduce the impact of different anaesthetics on immune function, total intravenous anaesthesia was used for the induction and maintenance of anaesthesia in this study. Upon entering the operating room, the patient received electrocardiogram monitoring and was administered propofol, cisatracurium, and sufentanil to maintain a BIS of 40–60. During surgery, patients in the naloxone group continued to use the PCIA pump to inject 0.05 mg/kg^-1^.h^-1^naloxone. Both groups of patients used PCIA pumps to control postoperative pain at the end of the surgery. Sufentanil 0.04 µg/kg^-1^.h^-1^ and saline diluted to 100 mL were used in the nonnaloxone group. In contrast, 0.05 µg/kg^-1^.h^-1^ naloxone was added to the naloxone group.

The PCIA pump had a total volume of 100 ml, with a background dose of 2 mL/h, a single dose of 0.5 mL, a locking time of 15 min, and a maximum of 20 mL/hour. At 50 h after surgery, when both sets of PCIA pumps were exhausted, naloxone treatment replaced the PCIA pump; 0.05 mg/kg^-1^.h^-1^ naloxone was administered for another 72 h. The nonnaloxone group did not receive any special treatment. The number of PCIA pump compressions and the degree of postoperative pain (evaluated by the VAS) were recorded.

The immune function of the two groups of patients was measured using the following methods: an electronic medical records system was used to collect routine peripheral blood from patients before surgery, one day after surgery, 4 days after surgery, and 6 days after surgery. The lymphocyte count, white blood cell count, and monocyte percentage were determined from routine blood test results. Using the same method, we extracted data related to patients’ peripheral blood calcitonin and IL-6 levels. We also recorded the patient’s hospitalization days, infection (abdomen, lungs), anastomotic fistula, fat liquefaction, and nausea/vomiting rate (recorded on the fourth day after the operation).

### Lymphocyte subset assay

Peripheral blood samples were collected from patients before surgery, one day after surgery, four days after surgery, and six days after surgery. Flow cytometry was used to analyse blood samples for lymphocyte subsets, and NK cells, B cells, and CD3 ^+^ T and CD4^+^/CD8 ^+^ T cells were extracted.

### TLR4, IL-6, and TNF-α assays

The collection of gastric specimens for scientific research was performed during surgery. Three gastric cancer tissues were selected and stored in liquid nitrogen. Next, QT-PCR technology was used to determine the levels of TLR4, IL-6, and TNF-α in the tissues.

### Statistical analysis

The purpose of this study was to examine changes in immune function, the incidence of perioperative complications, and the changes in TLR4 in gastric cancer tissue in two groups, (the naloxone group and the nonnaloxone group) SPSS 27.0 statistical software was used for statistical processing and analysis. The results are expressed herein as the means ± SDs or as numbers and percentages of participants, as appropriate. The measurement data were compared between groups using an independent sample t test. Repeated measures analysis of variance was used for intragroup comparisons at different time points; counting data are expressed as percentages, and intergroup comparisons were made using χ2 tests. Nonnormally distributed variables were analysed with the Mann‒Whitney U test. Differences were considered statistically significant at *P* < 0.05.

## Results

**Fig. 1:**
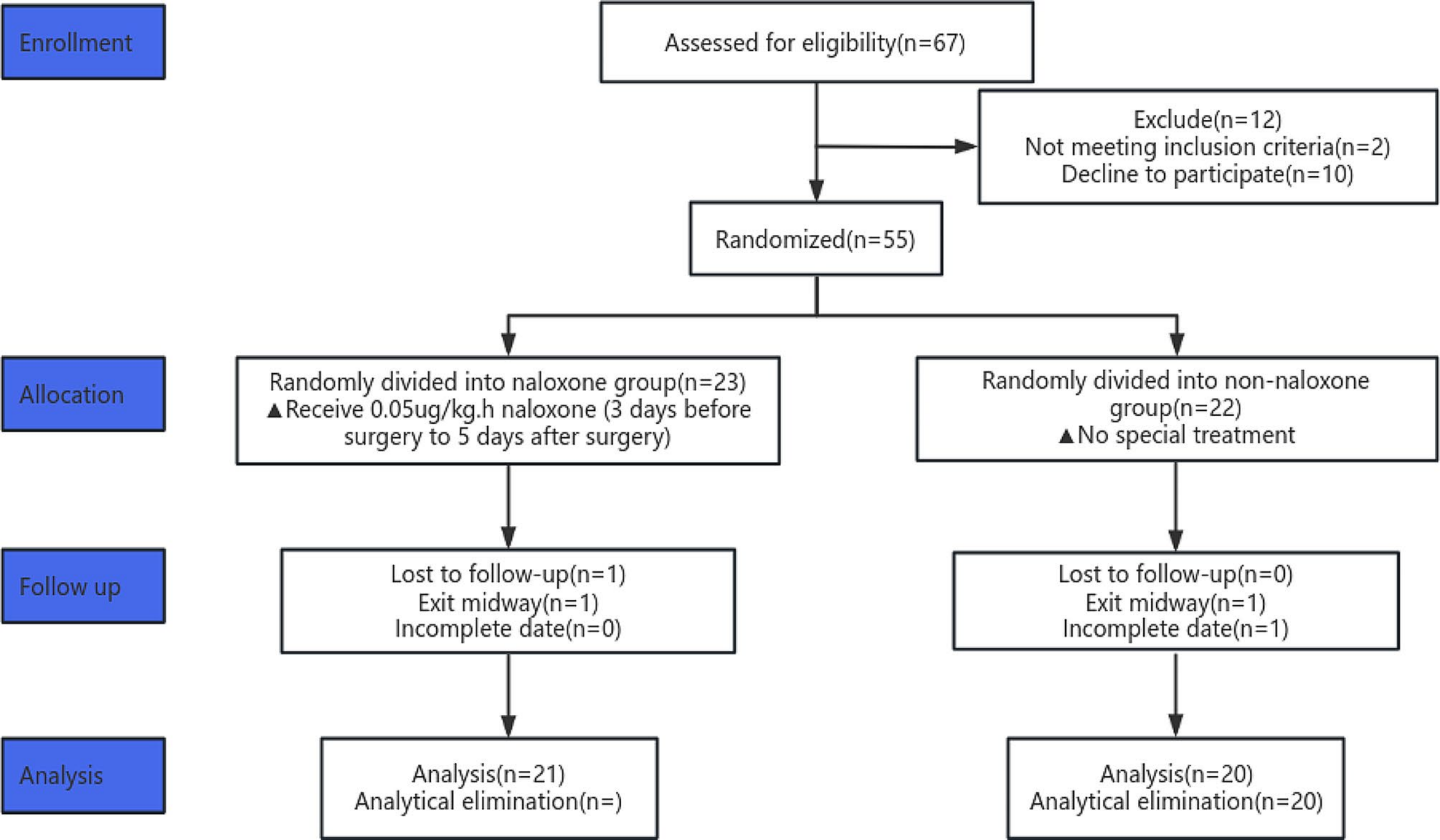
Flow chart of the study participants

Sixty-seven patients were initially enrolled; 2 patients did not meet the enrolment criteria, and 10 patients refused to participate in this study. The remaining 55 patients were randomly divided into a naloxone group and a nonnaloxone group. Three participants were excluded because of loss to follow-up (midway exit) or incomplete data (the patient was transferred to the ICU due to postoperative complications).The remaining 41 patients participated in the final data analysis (21 patients in the naloxone group and 20 patients in the nonnaloxone group) (Fig. 1). Neither group differed significantly in terms of continuous variables (age, duration of operation, weight, sufentanil dosage, BMI, NRS (nutrition risk screening)), TNM classification (tumour node metastasis classification) or categorical variables (ASA physical status, sex, and neoadjuvant chemoradiotherapy; Table 1).

**Table 1:** Patient characteristics

	Naloxone	Nonnaloxone	*t* value	*P* value
Age (years)	63.5 ± 6.3	64±5.8	0.60	0.55
Weight (kg)	67.3 ± 7.1	65.5 ± 9.7	0.68	0.50
BMI (kg/m^2^)	22 ± 2.4	22 ± 2.5	0.40	0.69
Duration of operation (min)	179.9 ± 13.5	178.5 ± 17.7	0.34	0.76
sulfentanil dosage (ug)	57.2 ± 7.8	56.5 ± 6.9	0.32	0.75
NRS	2.33 ± 0.97	2.30 ± 1.17	0.10	0.92
TNM classification				
T	1.81 ± 0.68	1.85 ± 0.75	0.18	0.86
N	1.43 ± 0.60	1.45 ± 0.91	0.12	0.90
M	0.09 ± 0.30	0.10 ± 0.31	0.05	0.96
Gender (case%)			χ^2 −^value	*p* value
Male	18	17	0.04	0.95
Female	3	3		
ASA physical status			0.5	0.48
II	16	17		
III	5	3		
Neoadjuvant chemoradiotherapy			0	1
yes	0	0		
No	21	20		

ASA American Society of Anaesthesiologists

**Fig. 2:**
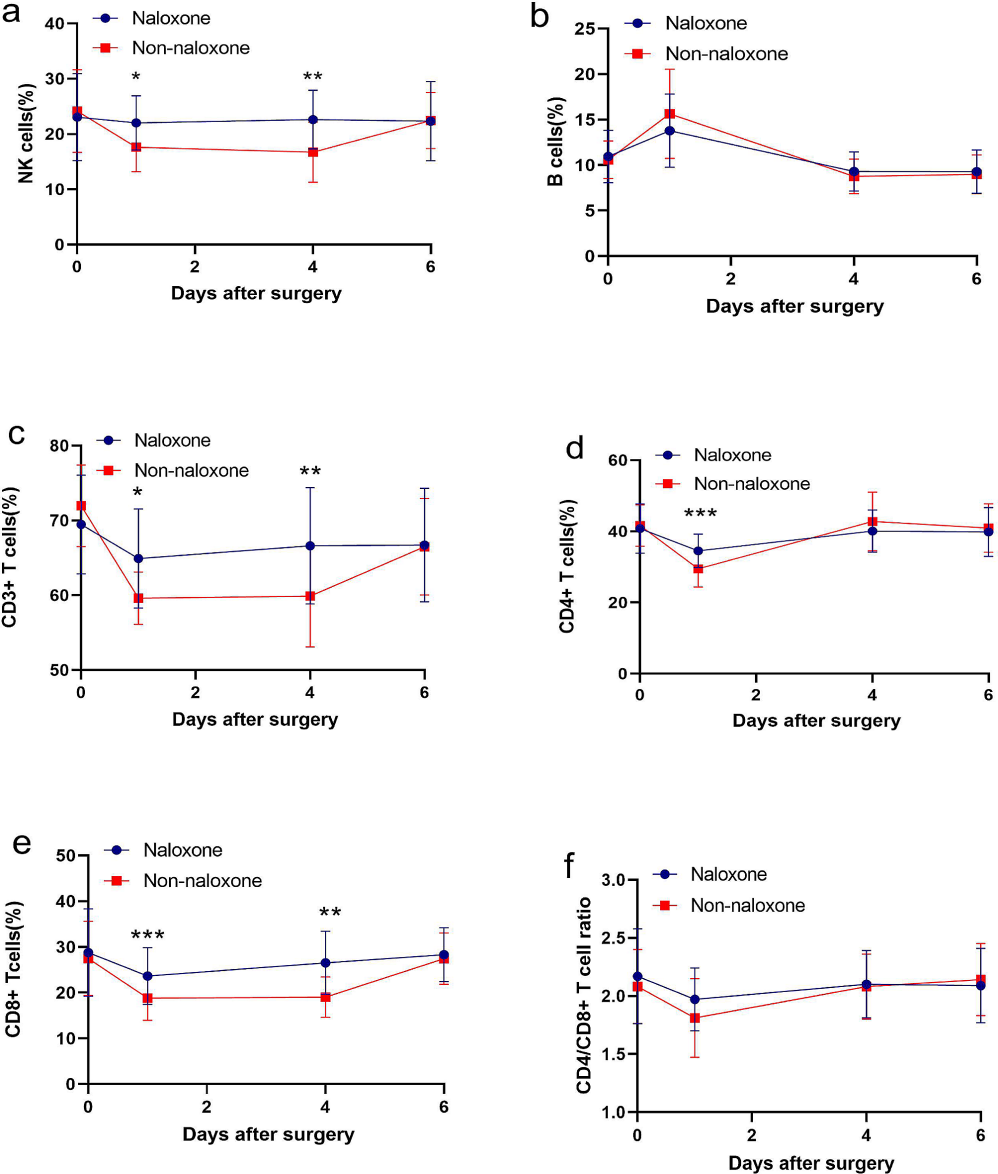
Comparison of peripheral blood lymphocyte subsets between the two groups. **a** Comparison of NK cells (%) between the naloxone and nonnaloxone groups (naloxone, *n* = 21. nonnaloxone, *n* = 20. *P < 0.05, **P < 0.01 vs. the nonnaloxone group). **b** Quantitative analysis of B cells (%) between the naloxone and nonnaloxone groups. **c** Quantitative analysis of CD3 ^+ ^T cells (%) in the naloxone and nonnaloxone groups (naloxone, *n* = 21). Nonnaloxone, *n* = 20. *P < 0.05, **P < 0.01 vs. the nonnaloxone group). **d** Quantitative analysis of CD4 ^+ ^T cells (%) between the naloxone and nonnaloxone groups (naloxone, *n* = 21). Nonnaloxone, *n* = 20. ***P < 0.001 vs. the nonnaloxone group). **e** Quantitative analysis of CD8 ^+ ^T cells (%) between the naloxone and nonnaloxone groups (naloxone, *n* = 21). Nonnaloxone, *n* = 20. **P < 0.01, ***P < 0.001 vs. the nonnaloxone group). **f** Quantitative analysis of the CD4^+^/CD8^ +^ T-cell ratio between the naloxone and nonnaloxone groups. (More data from Supplementary Table 1, Supplementary Table 2, Supplementary Tables 3 and Supplementary Table 4)

The nonnaloxone group showed a significant decrease in number of NK cells and CD8^+^ T cells at 24 h and 96 h after surgery compared with before surgery (*P* < 0.01) (Supplementary Table 4). At 24 h and 96 h after surgery, there were more NK cells and CD8^+^ T cells in the naloxone group than in the nonnaloxone group (*P* < 0.05) (Fig. 2). Compared with those before surgery, B cells in the naloxone group and nonnaloxone group showed a certain increase at 24 h after surgery and then returned to the presurgical level at 96 h after surgery (*P* < 0.05) (Supplementary Table 3, Supplementary Table 4). At 24 h after surgery, the nonnaloxone group exhibited a significant decrease in CD3^+^ T cells and CD4^+^ T cells compared to those in the naloxone group (Fig. 2). Compared with that before surgery, the CD4^+^/CD8 ^+^ T-cell ratio of the nonnaloxone group decreased at 24 h after surgery (Supplementary Table 4). There were no significant differences in the percentages of NK cells, B cells, CD3 ^+ ^T cells, CD4 ^+ ^T cells, or CD8^ +^ T cells or in the CD4^+^/CD8^ + ^T-cell ratio (Fig. 2) between the naloxone group and the nonnaloxone group 144 h after surgery.

Compared with those in the Naloxone group, WBC (*P* < 0.05) (Table 2) and neutrophil percentage (*P* < 0.01) (Table 2) were significantly greater in the Nonnaloxone group at 24 h after surgery and 96 h after surgery (*P* < 0.01) (Table 2). The WBC count (*P* < 0.05) (Table 2) and percentage of neutrophils in the nonnaloxone group were significantly greater at 24 h after surgery and 96 h after surgery (Table 2). There were no significant differences in the WBC count or neutrophil percentage between the groups at 144 h after surgery.

**Table 2:** Changes in white blood cell counts and neutrophil percentages in completed blood counts

		Naloxone	Nonnaloxone	*t* value	*P* value
WBC count/uL	Before surgery	5.74 ± 1.20	5.67 ± 0.68	0.24	0.81
	24 h after surgery	7.54 ± 1.73###	10.77 ± 1.97###	5.5	0.00
	96 h after surgery	5.75 ± 1.26	7.86 ± 1.44###	4.9	0.00
	144 h after surgery	6.23 ± 1.61	6.61 ± 1.85	0.67	0.49
Neutrophil Percentage (%)	Before surgery	61.24 ± 6.52	61.27 ± 7.59	0.01	0.99
	24 h after surgery	72.29 ± 5.71###	83.94 ± 3.37###	7.9	0.00
	96 h after surgery	66.19 ± 5.60	73.52 ± 6.86###	3.7	0.01
	144 h after surgery	62.6 ± 7.08	63.66 ± 6.51	0.75	0.46

# *P* < 0.05 versus “before surgery” for each group. ###*P* < 0.01 versus “before surgery” for each group. (More data from Supplementary Tables 5 and Supplementary Table 6)

The 2 groups showed significant increases in IL-6 and calcitonin (*P* < 0.01) (Table 3) at 24 h after surgery, 96 h after surgery (*P* < 0.01) (Table 3) and 144 h after surgery compared with the corresponding levels before surgery. The levels of IL-6 (*P* < 0.05) (Table 3) and calcitonin in the nonnaloxone group were significantly greater at 24 h after surgery, as shown in Table 3. There were no significant differences in the levels of IL-6 or calcitonin between the groups at 144 h after surgery.

Compared to the nonnaloxone group, the naloxone group had a lower incidence of infection (incision, abdomen, lungs) (*P* < 0.05) (Table 4). There were no significant differences in anastomotic fistula, nausea/vomiting, fat liquefaction or hospitalization days (days) between the groups.

**Fig. 3:**
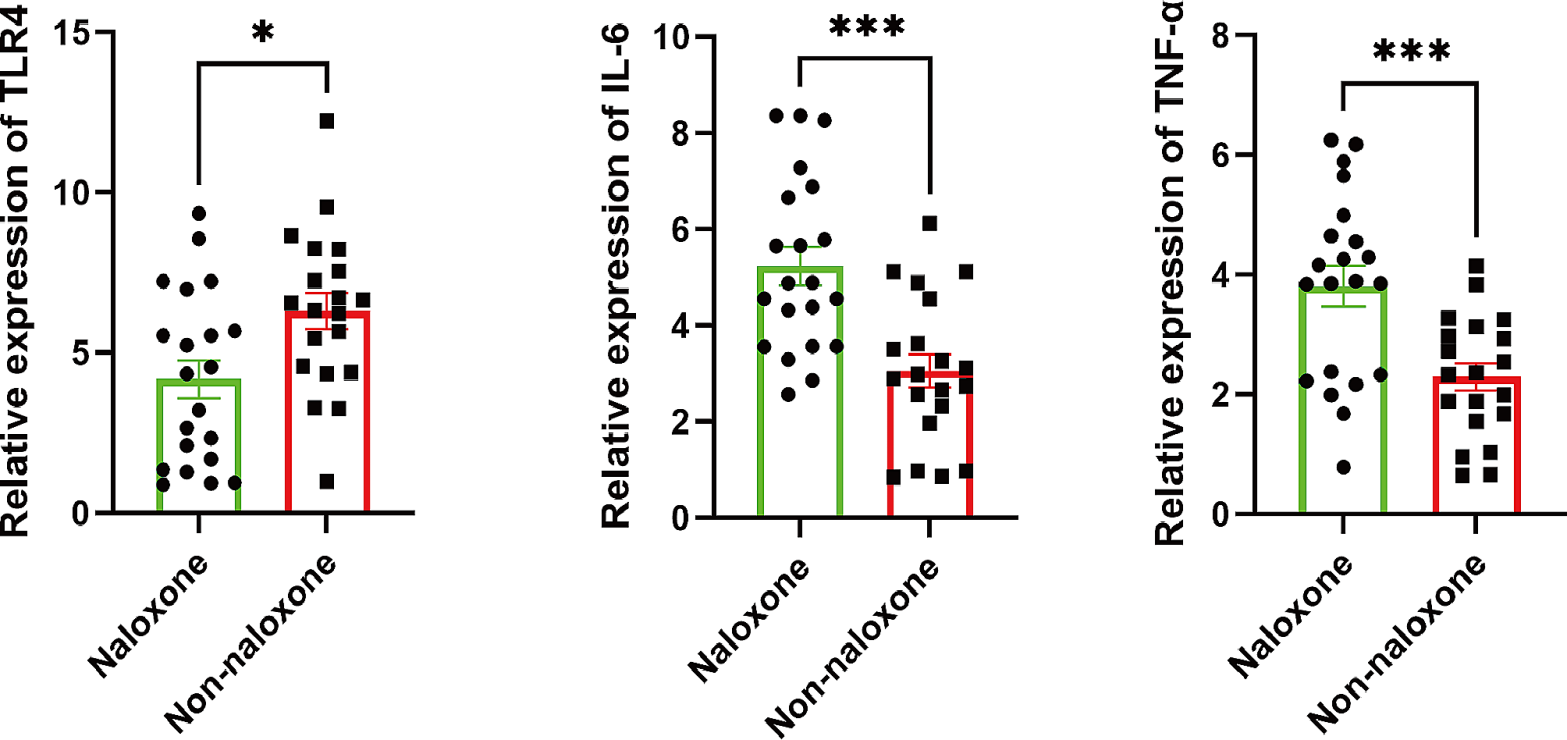
The expression levels of TLR4, IL-6 and TNF-α in gastric cancer tissue. The levels are presented as the means ± SDs. Significantly different from the nonnaloxone group at ***P < 0.001, *P < 0.05

Compared with those in the nonnaloxone group, the TLR4 levels were lower (*P* < 0.05) (Fig. 3) and the IL-6 and TNF-α levels were greater (Fig. 3) in the naloxone group.

**Fig. 4:**
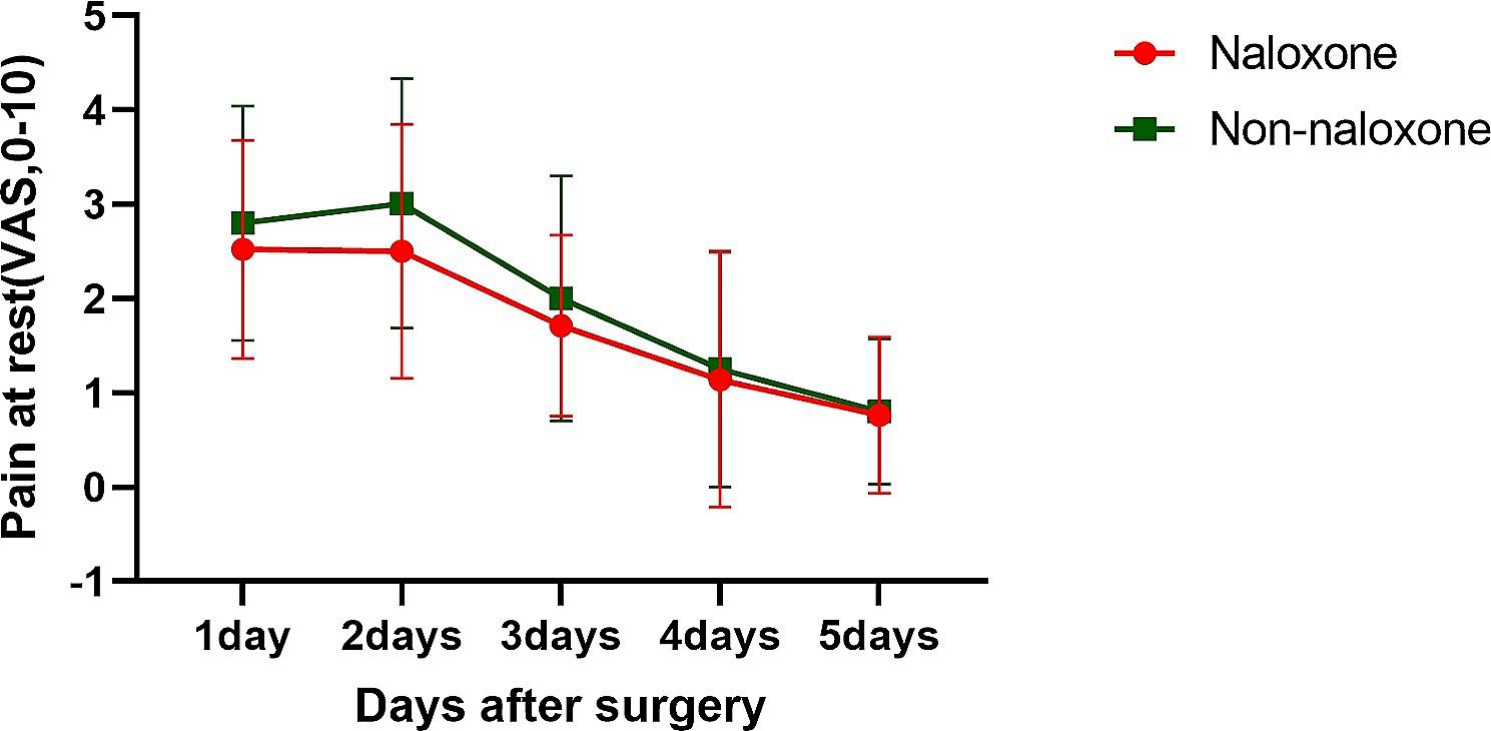
The visual analogue scale was used to assess the degree of pain at 1, 2, 3, 4, and 5 days after surgery. Data are presented as the means ± SDs

There were no significant differences in the VAS score between the groups at 1, 2, 3, 4, or 5 days after surgery (Fig. 4).

**Table 3:** Comparison of the serum IL-6 and calcitonin concentrations between the two groups

		Naloxone	Nonnaloxone	*t* value	*P* value
IL-6 (pg/ml)	Before surgery	5.48 ± 1.69	5.77 ± 1.59	0.56	0.58
	24 h after surgery	27.24 ± 8.70###	56.35 ± 16.35###	7.17	0.00
	96 h after surgery	17.43 ± 4.57###	25.69 ± 7.86###	4.10	0.00
	144 h after surgery	14.57 ± 4.73###	15.95 ± 3.27###	1.08	0.29
Calcitonin (ng/ml)	Before surgery	0.04 ± 0.02	0.05 ± 0.02	1.55	0.13
	24 h after surgery	1.05 ± 0.45###	1.53 ± 0.48###	3.25	0.02
	96 h after surgery	0.80 ± 0.27###	0.95 ± 0.32###	1.57	0.12
	144 h after surgery	0.39 ± 0.18###	0.41 ± 0.20###	0.25	0.81

# *P* < 0.05 versus “before surgery” for each group. ###*P* < 0.001 versus “before surgery” for each group. (More data from Supplementary Tables 5 and Supplementary Table 6)

**Table 4:** Comparison of postoperative complication rates between the two groups

Group	Number of cases	Infection (incision, abdomen, lungs) (%)	Anastomotic fistula (%)	Fat liquefaction (%)	nausea/vomiting (%)	Hospitalization days (days)
Naloxone	21	0	1	0	1	12.95 ± 1.72
Nonnaloxone	20	4	2	1	2	13.05 ± 1.93
χ^2 −^value		4.65	0.41	1.08	0.41	*t* value
						0.17
*P* value		0.03	0.52	0.30	0.30	0.87

## Discussion

According to the World Health Organization, stomach cancer is the fifth most common type of cancer in the world. China has the highest incidence of stomach cancer in the world. It is estimated that by 2024, there will be 1.77 million cases of stomach cancer worldwide [14]. The 5-year survival rate of gastric cancer patients is gradually improving with the advancement of treatment methods, such as chemotherapy, radiation therapy, targeted therapy, and immunotherapy. However, to date, surgery is still the only radical treatment method for gastric cancer. After laparoscopic total gastrectomy, patients may experience a decrease in their immune function due to mechanical damage to the lungs, and prolonged pneumoperitoneum, all of which may have adverse effects on their immune function. Preoperative neoadjuvant chemotherapy and tumours inhibit patients’ endogenous immune system activity, and the stress response caused by surgical trauma causes the body to release an excessive amount of inflammatory mediators. As a result, such patients experience more perioperative complications, are charged higher hospitalization costs, and are hospitalized for longer periods of time [15].

Naloxone has a structure similar to that of morphine and can specifically antagonize opioid receptors. It can be used for opioid overdose or alcohol at the conventional dosage of 50 mg/day. For cancer patients, different doses of naloxone may have different effects. Research has shown that continuous blocking of the OGF-OGFr axis caused by high doses of naloxone accelerates tumour growth, while intermittent blockade caused by LDN inhibits tumour growth. Currently, 5 mg/day of LDN is commonly used for antitumour therapy and treatment of autoimmune diseases [16, 17]. In addition, 0.05 mg/kg^− 1^.h^− 1^ naloxone has been shown to reduce the incidence and severity of perioperative nausea, vomiting, and pain [18], and this dose could also reduce postoperative complications of lung cancer surgery by modulating the function of immune cells, such as CD4^+^/CD8^+^ T-cell ratios and macrophages [19]. This is also the reason why the dose of naloxone used in this study was 0.05 µg/kg-1/h^− 1^ (3 days before surgery to 5 days after surgery).

Peripheral blood lymphocyte subpopulations are important indicators for measuring the immune function status of the body, with the most important being the detection of the levels of T cells, B cells, and NK cells [20]. NK cells are a crucial component of innate immunity and respond faster to infected cells or tumour cells than to T cells. A study by Na HY [21] indicated that the level of NK cells is strongly correlated with postoperative complications in gastric cancer patients. After activation, CD8 ^+ ^T cells differentiate into cytotoxic T cells, which secrete many cytokines. These cytokines, together with NK cells, form a crucial line of defence for the body’s antiviral and antitumour immunity. In this study, we found that compared with those before surgery, NK cells and CD8 ^+^ T cells were significantly lower in the nonnaloxone group at 24 h (*P* < 0.05) and 96 h after surgery (*P* < 0.01) (Supplementary Table 4). Similarly, the numbers of NK cells and CD8 ^+ ^T cells were significantly greater in the naloxone group than in the nonnaloxone group at 24 h (*P* < 0.05) and 96 h after surgery (*P* < 0.05) (Fig. 2). According to these findings, perioperative use of naloxone can reduce the decrease in NK cells and CD8 ^+ ^T cells in patients with gastric cancer following surgery. A greater density of NK cells and CD8 ^+^ T cells predicts a reduced incidence of postoperative complications in gastric cancer patients.

Different anaesthetic drugs exert immunoregulatory effects via different mechanisms. In vitro experiments have shown that high-dose opioids further decrease the production of cytokines (IL-2, IL-4, and IL-6) by inhibiting the proliferation of T cells, B cells, and NK cells [22]. Propofol, a sedative used for general anaesthesia, regulates autonomic nervous system tension during general anaesthesia, further improves perioperative immune function, and reduces cancer recurrence rates after surgery [23]. Unlike propofol, volatile anaesthetics have been shown to promote the growth of tumours [24, 25]. In this study, total intravenous anaesthesia was used for both the induction and maintenance of anaesthesia, and multiple types of anaesthetics were minimized during the anaesthesia process with the goal of reducing their impact on outcomes.

It is believed that CD3^+^ T cells represent the state of human cellular immune function and are a key indicator of both cellular immunity and the inflammatory response. CD4 ^+^ T-cell-inducible T cells/helper T cells are important hub cells for regulating the immune response. It is important to note that CD4^+^ T cells are inducible T cells/helper T cells that are important hub cells for regulating the immune response. The absolute count of CD4 ^+^ T cells usually fluctuates greatly depending on physiological conditions, while the ratio of CD4 ^+ ^T cells to CD8^+^ T cells is relatively stable. When the CD4 ^+ ^T-cell count is less than 200 and the CD4^+^/CD8 ^+^ T-cell ratio is less than 0.20, the likelihood of developing multiple opportunistic infections increases, as does the likelihood of multiple infections occurring simultaneously with the progression of the disease [26]. According to the statistical analysis of the results from this study, Naloxone had no significant effect on the number of B cells in gastric cancer patients, while in contrast with the naloxone group, the nonnaloxone group showed a significant decrease in CD3^+^ T cells and CD4^+^ T cells 24 h after surgery (Supplementary Table 3, Supplementary Table 4). The CD4^+^/CD8^+^ T-cell ratio in the nonnaloxone group decreased significantly after surgery (Fig. 2). According to these results, LDN slowed the decrease in CD3 ^+^ T cells and CD4^+^ T cells after gastric cancer treatment. The results of this study are consistent with the findings of Hamme’s [27] research, which indicates that LDN may exert an immunomodulatory effect by modifying the number of immune cells in lymphocyte subpopulations, thereby reducing postoperative complications in gastric cancer patients.

Tur-Martíne’s [28] findings suggest that systemic inflammation caused by the WBC count and neutrophil count has harmful effects on postoperative gastric cancer patients. An elevated leukocyte count is also a valuable indicator of stress response, wound infection, and lung infection. In addition, the WBC and neutrophil counts could also inhibit the antitumour effect of NK cells and cytotoxic T cells. We found that the nonnaloxone group had significantly greater WBC counts (*P* < 0.05) (Table 2) and neutrophil percentages (*P* < 0.01) (Table 2) at 24 h after surgery and 96 h after surgery (*P* < 0.01) (Table 2). The WBC counts (*P* < 0.05) (Table 2) and neutrophil percentages in the nonnaloxone group were significantly greater at 24 h after surgery and 96 h after surgery (Table 3). In other words, our perioperative application of LDN can reverse the postoperative increase in leukocytes in patients with gastric cancer. This can further reduce the incidence of postoperative complications in patients with gastric cancer.

IL-6, which is produced by lymphoid cells as well as certain nonlymphoid cells, such as macrophages, monocytes, dendritic cells, and mast cells, is one of the most common cytokines associated with inflammation. IL-6 plays an important role in host defence by regulating immune and inflammatory responses. Studies [29] have shown that LDN reduces the release of proinflammatory cytokines such as IL-6, IL-12 and tumour necrosis factor by blocking Toll-like receptors in T lymphocytes. Calcitonin is an important hormone in the human body that is mainly secreted by parafollicular cells of the thyroid gland. It can regulate the metabolism of calcium and phosphorus in the body and participate in the balance of calcium and phosphorus in the body. High calcitonin levels may be caused by diseases such as medullary thyroid cancer, small cell lung cancer, and medullary thyroid cancer, as well as by bacterial or fungal infections, allergies, and other reasons [30]. Our research revealed that the 2 groups exhibited significant increases in IL-6 (*P* < 0.01) (Table 4) and calcitonin 24 h after surgery, 96 h after surgery (*P* < 0.01) (Table 3) and 144 h after surgery. The levels of IL-6 (*P* < 0.05) (Table 3) and calcitonin in the nonnaloxone group were significantly greater at 24 h after surgery. These results indicate that perioperative naloxone use can delay the increase in the levels of IL-6 and calcitonin in postoperative gastric cancer patients. Our research results are consistent with those of Li, Meng-Yun et al. [31], who reported that perioperative administration of opioid antagonists suppressed the expression of proinflammatory cytokines, which resulted in the suppression of inflammatory reactions.

Postoperative complications are given a high degree of attention. In numerous studies [32, 33], LDN has been shown to significantly decrease pain intensity, opioid drug consumption, and opioid-induced nausea and pruritus following surgery. The purpose of this study, however, was to observe the complications caused by changes in immune function. The use of 0.05 µg/kg^-1^.h^-1^ naloxone during the perioperative period reduced the rate of postoperative infection complications (incision, abdomen, and lungs) among patients who underwent laparoscopic total gastroplasties (Table 4). For the first time, this article reports that 0.05 µg/kg^-1^.h^-1^ naloxone can reduce the incidence of postoperative complications associated with infection in patients undergoing laparoscopic total gastrectomy.

To explore the specific mechanism by which LDN reduces postoperative complications, we conducted scientific research on gastric cancer tissue and analysed the expression levels of TLR4, IL-6 and TNF-α. One study [34] demonstrated that LDN protects against the inflammatory response induced by autoimmune hepatitis, which may be related to its ability to suppress IL-6 and TNF-α secretion and interfere via modulation of the TLR4/NF-ƙB signalling pathways. Our research results indicate that LDN reduces the expression level of TLR4 in gastric cancer tissue and increases the expression level of IL-6 in gastric cancer tissue. This may be because LDN leads to the activation of IL-6 and TNF-α secretion by CD8 ^+ ^T cells through the TLR4/AKT/mTOR pathway [34]. However, its specific molecular biological mechanism needs to be further studied.

LDN has been shown to potentiate opioid analgesia and reduce postoperative pain [35]. Unfortunately, this study did not show that LDN improved postoperative VAS scores in patients with gastric cancer, possibly due to the different types of surgeries used in this study (total gastrectomy) compared to those used by other researchers (lobectomy). The results of this study indicate that 0.05 µg/kg^-1^.h^-1^ naloxone does not increase postoperative pain and can be safely used in patients undergoing laparoscopic total gastrectomy. This study also shows that LDH does not reduce the incidence of postoperative nausea and vomiting in patients. This finding is inconsistent with the results of some researchers [36], which may be due to the impact of different surgical methods on the research results.

There are four limitations in this study. First, the long-term recurrence rate of tumours was not observed. Studies indicate that LDN exerts its immunoregulatory effects by binding to opioid receptors in or on tumour cells and immune cells. In the treatment of cancer and other immune-related diseases, LDN may prove to be an effective immunomodulatory agent [37]. Second, naloxone was administered at a dosage of 0.05 mg/kg^-1^.h^-1^ in this study, and the impact of other concentrations of naloxone on postoperative gastric cancer patients needs to be investigated further. Third, in this study, only the changes in TLR4 in gastric cancer tissues were studied, and other proteins involved in signal transduction pathways were not studied. In the next step, we will design experiments to study the specific molecular biological mechanism involved. Fourth, research has shown that naloxone may improve patients’ immune function by affecting macrophage function [38]. However, this study did not investigate macrophage changes. Next, we will conduct animal experiments to explore the specific molecular and biological mechanisms involved.

## Conclusion

Overall, 0.05 ug/Kg^-1^.h^-1^ naloxone can reduce the rate of postoperative infection complications (incision, abdomen, and lungs) in patients who undergo laparoscopic total gastrectomy. This may be because at this dosage, naloxone alters the number of NK, CD3 ^+^ T, CD4 ^+^ T and CD4^+^/CD8 ^+^ T cells in the lymphocyte subpopulations or alters TLR4 receptor expression in immune cells and thus induces changes in their activity.

### Electronic supplementary material

Below is the link to the electronic supplementary material.

Supplementary Material 1

Supplementary Material 2

Supplementary Material 3

Supplementary Material 4

Supplementary Material 5

Supplementary Material 6

## Data Availability

No datasets were generated or analysed during the current study.

## Abbreviations

LDN: low-dose naloxone
ASA: American Society of Anaesthesiologists
CD: clusters of differentiation
OGF-OGFr: opioid growth factor-opioid growth factor receptor
TLR: Toll-like receptor
PCIA: patient-controlled intravenous analgesia
T cells: T lymphocytes
VAS: visual analogue scale
WBC: white blood cell
NRS: nutritional risk screening
TNM classification: (tumour node metastasis classification)
